# Genetic Variability in Polish Lowland Sheepdogs Assessed by Pedigree and Genomic Data

**DOI:** 10.3390/ani10091520

**Published:** 2020-08-27

**Authors:** Paula Wiebke Michels, Ottmar Distl

**Affiliations:** Institute of Animal Breeding and Genetics, University of Veterinary Medicine Hannover (Foundation), 30559 Hannover, Germany; paula.wiebke.michels@tiho-hannover.de

**Keywords:** genetic diversity, inbreeding, demography, pedigree analysis, single nucleotide polymorphisms, runs of homozygosity, dog

## Abstract

**Simple Summary:**

Dogs are an important part of society. The Polish Lowland Sheepdog (PON) is one of 353 of the world’s largest cynological organization listed dog breeds. Breeds with small population sizes, like the PONs, are often characterized by high inbreeding rates and thus an increased risk of congenital diseases. To examine the endangerment of the PONs, measures for genetic diversity and inbreeding were calculated for the German PON population. The study showed that the PONs had to be classified as a minimally endangered population according to threshold values specified by the European Association for Animal Production. However, the very recent trend showed a slight improvement.

**Abstract:**

Genetic variability of Polish Lowland Sheepdog (PON) population was evaluated using both pedigree and genomic data. The analyzed pedigree encompassed 8628 PONs, including 153 individuals genotyped on the Illumina CanineHD BeadChip. Runs of homozygosity (ROH) were defined for homozygous stretches extending over 60 to 4300 kb. The inbreeding coefficients *F_Ped_* based on pedigree data and *F_ROH50_* based on ROHs were at 0.18 and 0.31. The correlation between both was 0.41 but 0.52 when excluding animals with less than seven complete generations. The realized effective population size ($\bar{N_{e}}$) was 22.2 with an increasing trend over years. Five PONs explained 79% of the genetic diversity of the reference population. The effective population size derived from linkage disequilibrium measured by r^²^ was 36. PANTHER analysis of genes in ROHs shared by ≥50% of the PONs revealed four highly over- or underrepresented biological processes. One among those is the 7.35 fold enriched “forelimb morphogenesis”. Candidate loci for hip dysplasia and patent ductus arteriosus were discovered in frequently shared ROHs. In conclusion, the inbreeding measures of the PONs were high and the genetic variability small compared to various dog breeds. Regarding $\bar{N_{e}}$, PON population was minimally endangered according to the European Association for Animal Production.

## 1. Introduction

The calculation of demographic measures and thereby the evaluating of the genomic architecture of a breed are an important issue in dog breeding. Pedigree-based inbreeding coefficients in several dog breeds ranged from 0.03 to 0.1 [1,2,3]. The estimation of genetic variability has been both performed based on pedigree and single nucleotide polymorphism (SNP) array data. However, genome-based measures are more accurate than pedigree-based [4] and inbreeding coefficients based on genomic data were higher compared to those based on pedigree data [3].

The genetic variability largely depends on breeding decisions and practices. Selection for special characteristics in dog breeds can result in bottlenecks in the populations and thus high inbreeding rates. Thereby, the probability of homozygosity for recessive genes increases, which leads to a higher incidence of hereditary diseases [5]. Especially in small populations, where the possible choice of breeding mates is small, the risk of highly inbred offspring increases.

Polish Lowland Sheepdogs (Polski Owczarek Nizinny, PON) are an example for a small population. In 2009 to 2018 only 57 to 116 PONs per year were born in the German Kennel Club (VDH) [6]. In contrast, over all breeds the average of dogs born per year in the VDH was 255 to 299, with a range from 0 to 15,870, in this period [6]. At the beginning of pure breeding after World War I PONs were highly inbred on one dog to introduce a breeding standard [7]. The Fédération Cynologique Internationale (FCI) officially recognized the medium-sized dogs in 1963 under the standard number 251 [8]. The breed originally is a sheepdog, but nowadays most often a pet. An inbreeding coefficient (*F_Ped_*) of 0.04 was calculated for an Australian PON population [9]. However, the studied population contained only 12 animals. As summarized by Bell, et al. [10] health problems in PONs are: Canine hip dysplasia (CHD), hypothyroidism, several ocular diseases like progressive retinal atrophy (PRA) and cataract, neuronal ceroid lipofuscinosis, and patent ductus arteriosus (PDA). According to the disease statistics of the Orthopedic Foundation for Animals the most frequent diseases are CHD and PRA *(rcd4*-mutation) with 16.7% (of 634 dogs examined dogs) and 17.5% (of 331 dogs) abnormal results [11]. The *rcd4*-mutation explained most of the PRA cases in PONs [12]. A few cases (1.6%/127) of canine elbow dysplasia (CED) were also reported in the OFA statistics [11].

The objective of this study was to analyze the genetic variability of the PONs registered in the Allgemeiner Klub für polnische Hunderassen e.V. (APH), Germany. The measures were estimated using pedigree and SNP array data. Therefore, 153 PONs were genotyped on the Illumina CanineHD BeadChip (Illumina, San Diego, CA, USA). The genetic variability was estimated based on linkage disequilibria (LDs) and runs of homozygosity (ROHs). Estimated fixation index *F_IS_* and *F_Ped_* based on pedigree data were compared to *F_IS_* and *F_ROH_* based on genotype information. The genes located in the ROHs were considered regarding their potential relevance for potential health problems of PONs.

## 2. Materials and Methods

### 2.1. Data, Samples, and Genotyping

Pedigree data were provided by the APH. Missing information in pedigrees was supplemented with data from herd books. The pedigrees encompassed 8628 PONs born since 1953 and with parental information. Blood samples of 153 PONs were obtained from the bio-bank of the Institute for Animal Breeding and Genetics at the University of Veterinary Medicine Hannover, which contained blood samples of about 800 APH-registered PONs. Sampling of the genotyped PONs regarded population structure and was done in such a way that sires and dams with larger progeny numbers were represented in the data set. These animals were born from 1995 to 2017. Genomic DNA was extracted using a standard ethanol fractionation with concentrated sodiumchloride (6 M NaCl) and sodium dodecyl sulphate (10% SDS). The concentration of each DNA sample was adjusted to 50 ng/µL and then genotyped using the Illumina CanineHD BeadChip (Illumina, San Diego, CA, USA) containing 220,853 SNPs.

### 2.2. Analysis of Pedigree Data

**Figure 1:**
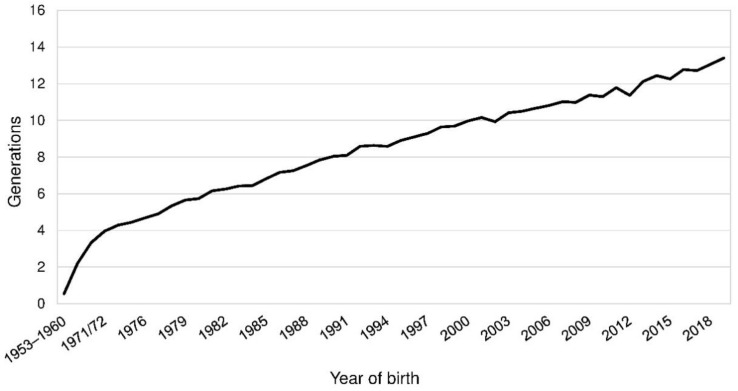
Mean equivalent complete generations by birth years of Polish Lowland Sheepdogs (PONs). The mean equivalent complete generations across all 8628 PONs was 8.73 generations.

The demographic measures based on pedigree data were estimated using the software ENDOG, version 4.8, Departamento de Producción Animal, Facultad de Vaterinaria, Universidad Complutense de Madrid, Madrid, Spain [13]. All parameters were calculated for a reference population and the BeadChip sample, which contained the 153 genotyped PONs with available pedigree information. Both were considered as subpopulations of the entire APH-registered PON population. The reference population was chosen on basis of the pedigree completeness. For each birth cohort the mean equivalent complete generations (EqG) were calculated (Figure 1). To reach a number of at least 30 animals per cohort those from 1953 to 1968 were put into bins of eight years and those from 1969 to 1974 into bins of two years. EqG are the sum over all known ancestors [14]. The parameter was calculated as the sum of (1/2)*^n^* where *n* was defined as the number of generations separating the individual to each known ancestor. The threshold for birth cohorts to be included in the reference population was set at EqG larger than eight. Dogs born in 2019 were excluded because of the incomplete information of the cohort. Thus, the reference population included all PONs born from 1990 to 2018 dogs. These were 4920 PONs. Per year, 91 to 397 PONs were born (Figure S1). The BeadChip sample was a part of the reference sample and for this subpopulation genealogical and genomic measures were compared.

Further measures of pedigree completeness were the completeness of each ancestor in the pedigree to the 5th parental generation and the index of completeness by MacCluer, et al. [15].

The generation interval was computed as the average age of the parents at the birth of their offspring that was used for reproduction [16].

As one measure of genetic variability the effective number of founders (*f_e_*) was calculated as

(1) $$f_{e}=\frac{1}{\sum_{k=1}^{f}q_{k}^{2}}$$

where *f* was the number of founders and *q_k_* the probability of gene origin of the individual ancestor (*k*) [17]. The parameter is defined as the number of equally contributing founders that are expected to produce the same genetic diversity as in the population under study [18]. Founders are those animals without known ancestors and to which all other animals of the population can be traced back.

To evaluate the balanced use of reproductive individuals in the PON population we computed the effective number of ancestors (*f_a_*). The parameter was calculated as the marginal genetic contribution (*q*) of an individual ancestor (*q_j_*), which is the contribution by an ancestor that could not be explained by other ancestors before:

(2) $$f_{a}=\frac{1}{\sum_{j=1}^{a}q_{j}^{2}}$$

with *a* = number of ancestors [17].

Founder genome equivalents (*N_g_*) are the number of equally contributed founders that would be expected to produce the same genetic diversity as the population under study without any random loss of founder alleles in descendants [18]. The program segreg.f of the PEDIG software, Institut National de la Recherche Agronomique, Jouy en Josas Cedex, France [19] was used for the calculations.

To evaluate the unbalanced use of reproducers, we calculated the effective number of sires (*NeffS*) and dams (*NeffD*) as:

(3) $$NeffS=\;\frac{1}{\sum_{i}s_{i}{}^{2}}$$

(4) $$NeffD=\;\frac{1}{\sum_{i}d_{i}{}^{2}}$$

where *s_i_* (*di*) was the relative frequency of use of the sire or dam *i* among all sires (dams) of the reference population [20].

Furthermore, the inbreeding coefficient (*F_Ped_*) according to Meuwissen and Luo [21] was calculated. The parameter describes the probability that an individual is homozygous by descent at a random chosen locus in the genome.

We estimated *F_IS_* as a part of the F-Statistics [22]. It was calculated with

(5) $$F_{IS\_Ped}=\frac{\tilde{F_{Ped\;}}-\;\bar{f}}{1\;-\;\bar{f}}$$

where $\tilde{F_{Ped}}$ was the mean inbreeding coefficient for the PON population and $\bar{f}$ the mean coancestry for the subpopulation [23,24]. We defined every birth cohort as one subpopulation.

The individual increase in inbreeding (Δ*F_Ped_i_*) was calculated based on *F_Ped___i_* dependent on the equivalent complete generations (*t*) [25]:

(6) $$\Delta F_{Ped\_i}=1\;-\sqrt[{\;t\;-\;1}]{1-F_{Ped\_i}}.$$

Thereof the increase in inbreeding of the reference population (Δ*F_Ped_*) was calculated as mean of the individual values.

The effective population size was estimated as realized effective size ($\bar{N_{e}}$) from the individual increase in inbreeding with [26]:

(7) $$\bar{N_{e}}=\;\frac{1}{2\bar{\Delta F_{Ped}}}$$

To get an even more precise *N_e_* in the case of substructures in the studied population [27], the *N_e_* additionally was estimated via the paired increase in coancestry according to [26]:

(8) $$\Delta c_{jk}=1\;-\;\sqrt[{\left(\frac{g_{j}\;+\;g_{k}}{2}\right)}]{1\;-\;c_{jk}}$$

where *c_jk_* was the coancestry coefficient between the two paired individuals *j* and *k* and *g_j_* and *g_k_* the discrete equivalent generations of *j* and *k*.

The average coancestries of the reference population and the BeadChip sample were calculated using the program *par3.f* of the PEDIG software [19].

### 2.3. Statistical Analysis of Genomic Data

For the estimation of the genetic variability measures, all SNPs from sex chromosomes were excluded from the dataset. SNPs and individuals with a genotyping rate < 0.95 were removed. We did not remove SNPs through MAF to avoid a removing of homozygous regions. Previously, in a similar study a filtering for MAF did mostly result in indistinguishable estimates [28]. Thus, the final dataset contained 167,183 autosomal SNPs. These SNPs covered 2,201,666,442 bp of the autosomal dog genome.

PLINK (www.cog-genomics.org/plink/1.9/), version 1.9, Complete Genomics, Mountain View, CA, USA [29] was used for the ROH detection. The minimum run length of SNPs in a ROH *(l*) resulting in a type I error rate *α* of 0.05 was calculated as:

(9) $$l\;=\frac{\log\left(\frac{\alpha }{n_{SNP}\;\times \;n_{i}}\right)}{\log\left(hom\right)}$$

where *n_SNP_* was the mean number of SNPs per individual, *n_i_* the number of genotyped individuals and *hom* the mean homozygosity across all SNPs [30,31]. This corresponded to a length of 50 SNPs. We defined a ROH as a homozygous stretch that extended over 60 to 4300 kb and according to the average SNP distance on the array contained at least 5, 7, 10, 20, 30, 40, 50, 65, or 358 SNPs, respectively (Table 1) [30,31,32]. It has been previously shown that selection signatures going back many generations could only be captured in short ROHs with a type I error rate *α* > 0.05 [33]. Especially in regions with low marker density or high recombination rates use of smaller ROHs had benefits [34]. Therefore, the short ROHs containing less than 50 SNPs were included in the study. In order to capture recent inbreeding, ROHs containing more SNPs were included in the study. We did not allow for heterozygous SNPs and only two to five missing SNP genotypes in the homozygous region were admitted [32]. The matching proportions of overlapping ROHs were pooled. A consensus ROH was a matching proportion that occurred in all PONs. We watched out for known causal or associated loci and genes for the most breed-relevant diseases CHD and PRA (*rcd4*-mutation) located in the regions of the *ROH5* shared by at least 50, 75, or 90% of the genotyped PONs. Furthermore, we screened for genes *fibroblast growth factor 5* (*FGF5*) and *R-spondin 2* (*RSPO2*) responsible for the coat structure of the PONs as all PONs are long coated and have furnishings according to the breeding standard [35]. Screening was done for *ROH5* to cover a maximum number of ROHs including the very ancient ones. Significant differences of the amount of ROHs per chromosome were tested with a paired samples Wilcoxon test and R, version 3.6.3, R Foundation for Statistical Computing, Vienna, Austria [36].

The inbreeding coefficient *F_ROH_* and fixation index *F_IS_SNP_* were estimated using the software SAS, version 9.4 (Statistical Analysis System, Cary, NC, USA, 2019). *F_ROH_* was calculated as a proportion of the length of all ROHs to the total length of all autosomes covered by SNPs [37]. *F_IS_*-values for each individual (*i*) were calculated as:

(10) $$F_{IS\_SNPg,i}=\;\frac{O_{i}-\;E_{i}}{n_{SNP,i}-\;E_{i}},$$

with *E_i_* = number of expected homozygous SNPs by chance, *O_i_* = number of observed homozygous SNPs and *n_SNP,i_* = number of all observed autosomal SNPs in the considered individual [38]. Pearson correlation coefficients for the inbreeding coefficients were calculated. To prove the impact of the complete generations on the correlation between *F_Ped_* and *F_ROH_*, dogs with less than four, five, six, and seven, respectively, complete generations in the pedigree dataset were excluded from the calculations.

The effective population size and the increase in inbreeding per generation based on the LD were detected using PLINK (www.cog-genomics.org/plink/1.9/), version 1.9 [29]. Therefore, the LD was measured as the squared correlation between SNP pairs (*r*^2^). Distance bins of 0.1 Mb were created containing the *r^²^*-values for SNP pairs with distances of 1 kb to 33.3 Mb. For each distance bin the mean *r^²^*-value was estimated. The *N_e_SNP_* was computed as $N_{e\_SNP}=\;\frac{1-\;r^{2}}{4cr^{2}}$, with *c* = recombination rate in Morgan units [39]. Regarding the distance *c* we assumed that 100 Mb = 1 Morgan. Δ*F_SNP_* was estimated with $\Delta F_{SNP}=\;\frac{1}{2N_{e\_SNP}}$ [27]. The number of generations in the past (*t*) was computed as $t=\;\frac{1}{2c}$ and rounded to the nearest integer. If there was more than one value of *N_e_SNP_* or Δ*F_SNP_* per integer, the values were averaged.

Using Net View package, version 3.4.1 [40,41] implemented in R, version 3.6.3 [36], a NetView analysis was applied to detect fine-scale population structures. It is based on a relationship matrix which was derived from distances between SNP markers calculated with PLINK (www.cog-genomics.org/plink/1.9/), version 1.9 [29,41]. NetView clusters based on mutual k-nearest neighbors were created to visualize the relatedness between the individuals [40,41]. The maximum number of nearest neighbors (K-NN) was set to K-NN = 2–10 as recommended for small animal sets [41]. Analyses at higher K-NN to investigate large-scale genetic structures were not performed, because the analysis just included one breed. The visualization of the population network for K-NN = 7 and 10 was done using Cytoscape, version 3.7.0 [42]. To enable a visualization of potential connections between genetic distances and inbreeding coefficients, the PONs were grouped into classes of different inbreeding coefficients (*F_ROH5_* < 0.16, *F_ROH5_* = 0.16–0.19, *F_ROH5_* = 0.19–0.22, *F_ROH5_* = 0.22–0.25, *F_ROH5_* = 0.25–0.28, *F_ROH5_* = 0.28–0.31, *F_ROH5_* = 0.31–0.34, *F_ROH5_* = 0.34–0.37, *F_ROH5_* = 0.37–0.40, *F_ROH5_* = 0.40–0.43, *F_ROH5_* ≥ 0.43).

PANTHER 15.0 (Protein Analysis Through Evolutionary Relationships), Division of Bioinformatics, Department of Preventive Medicine, Keck School of Medicine of USC, University of Southern California, Los Angeles, CA, USA [43] was used to investigate the functional relevance of the ROHs. All genes located in *ROH5* shared by at least 50, 75, or 90% of the PONs were analyzed by the “functional classification” analysis and the “statistical overrepresentation” test. The overrepresentation test was done for the annotation sets “GO biological process complete” and “Panther Protein Class”. To cover maximal ROHs including the very ancient ones the analysis was started for *ROH5*.

In addition, the ROHs shared by 75 or 90% of the PONs were cross-referenced with the databases OMIM [44] and OMIA [45].

The datasets used and/or analyzed during the current study are available from the corresponding author on reasonable request.

## 3. Results

### 3.1. Demographic Measures Using Pedigree Analyses

**Figure 2:**
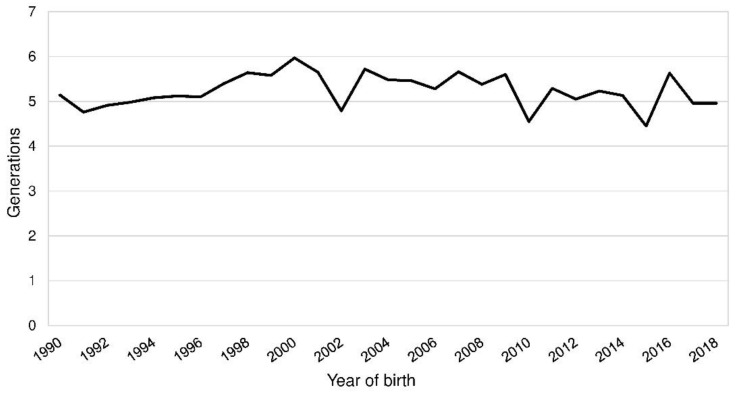
Mean complete generations by the year of birth of Polish Lowland Sheepdogs (PONs).

The demographic measures were calculated for a reference population born from 1990 to 2018 and the BeadChip sample, which contained the 153 genotyped PONs (Table 2; Table S1). The mean of the EqG in the reference population was 10.1 and 11.2 in the BeadChip sample. Complete generations varied from 4.5 to 6.0 in the reference population (Figure 2). In the fifth parental generation, 93.1 to 99.1% of the ancestors were known in the reference population (BeadChip: 86.3–100.0%). The index of completeness by MacCluer, Boyce, Dyke, Weitkamp, Pfenning, and Parsons [15] was above 0.9 up to the seventh and ninth generation in the reference population and the BeadChip sample.

The mean generation interval was 4.7 years in the reference population (BeadChip: 4.9 years). Ten effective founders and six effective ancestors were identified. Five of the ancestors explained 79% respectively 78% of the genetic diversity of the population in the reference population and the Beadchip sample. *NeffS* was 150 at a total number of 433 sires and *NeffD* was 335 at a total number of 700 dams.

**Figure 3:**
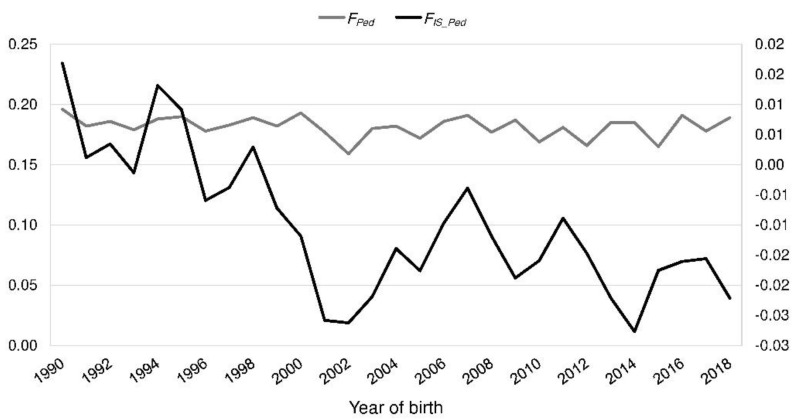
Inbreeding coefficient (*F_Ped_*) and fixation index *F_IS_Ped_* by the year of birth of the Polish Lowland Sheepdog reference population.

**Figure 4:**
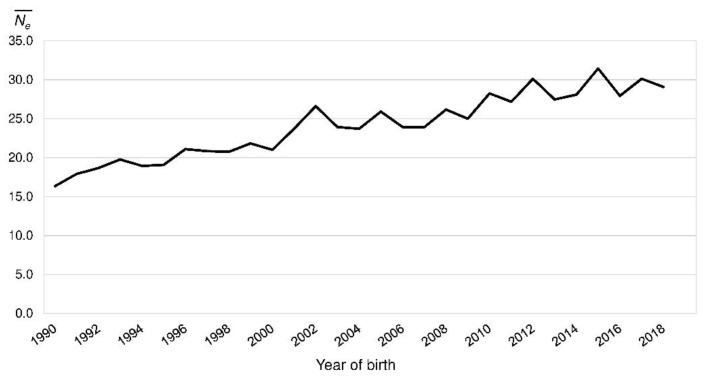
Realized effective population size ($\bar{N_{e}}$) by the year of birth of the Polish Lowland Sheepdog reference population.

In the reference population, *F_Ped_* ranged from 0.00 to 0.45 with a mean of 0.18 (BeadChip: 0.05–0.40, mean 0.18). It was consistent over the last 30 years (Figure 3). *F_IS_Ped_* decreased from 0.017 in 1990 to −0.022 in 2018 (Figure 3). The realized *N_e_* increased from 16.3 (1990) to 29.1 (2018) (Figure 4).

### 3.2. Measurement of Genetic Variability Using Genomic Data

**Figure 5:**
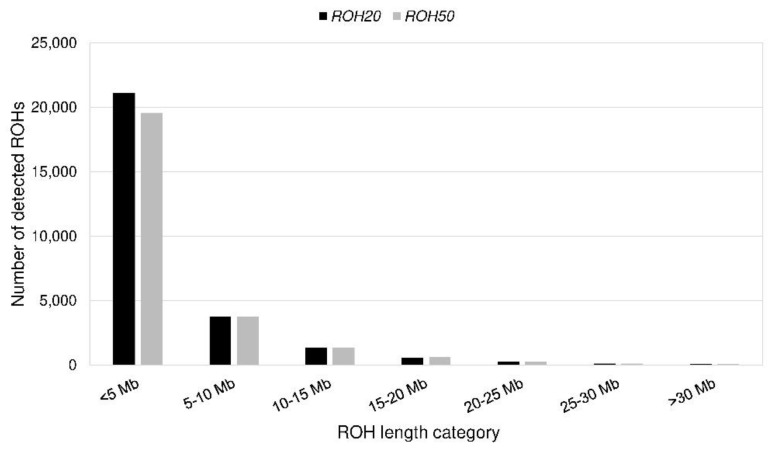
Number of detected runs of homozygosity (ROHs) per length category for *ROH20* and *ROH50*.

**Figure 6:**
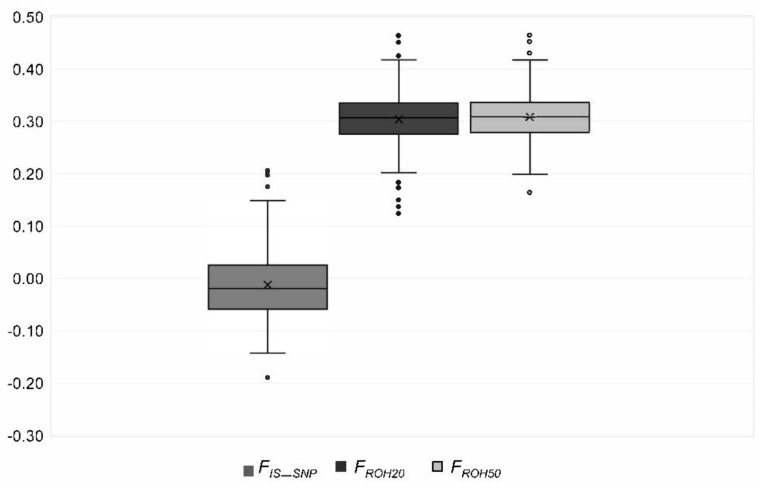
Distribution of the genomic inbreeding coefficients *F_ROH20_* and *F_ROH50_* and the fixation index *F_IS_SNP_* in the genotyped Polish Lowland Sheepdogs. Outliers are given with dots.

We identified 6764 (*ROH358*) to 27,276 (*ROH5*) ROHs (Table 3). None of the ROHs was a consensus ROH. Eight of the *ROH5* were shared by at least 75% of the PONs (Table 4). The length varied from 61 kb to 83 Mb with an average length of 3.8 ± 4.9 Mb (*ROH5*) to 10.4 ± 6.5 Mb (*ROH358*). Most ROHs (77%) had a length less than 5 Mb (Figure 5). On average, CFA 29 had the most homozygous regions (Figure S2, Table S2). The SNPs that were homozygous most frequent (99%) were located on CFA 13 (Figure S3). The mean *F_ROH_* for all genotyped PONs was 0.30 to 0.31, respectively, and ranged from 0.12 to 0.47 except *F_ROH358_*, which was 0.21 (0.00–0.41) (Table 3, Figure 6). For *F_IS_SNP_* a mean of −0.01 (−0.19 to 0.21) was calculated (Figure 6). The parameter was below zero in 62% of the PONs. There was a statistically significant (*p* < 0.05) correlation of 0.92 between *F_ROH_* and *F_IS_SNP_*. Between *F_ROH_* and *F_Ped_*, the Pearson correlation coefficient ranged from 0.25 (*F_ROH358_*) to 0.42 (*F_ROH40_*) with a *p*-value > 0.0001 (Table 5). An exclusion of dogs with low complete generations lead to an increase of the correlation (Table 6).

The gene for furnishing and quantitative trait loci previously reported to be associated with CHD, were located within the *ROH5* shared by at least 50, 75, and 90% of the PONs (Table S3) [46,47,48,49,50,51,52]. According to the database OMIM, the *gene angiopoietin 1* (*ANGPT1*) is involved in the development of vascular structures [53].

**Figure 7:**
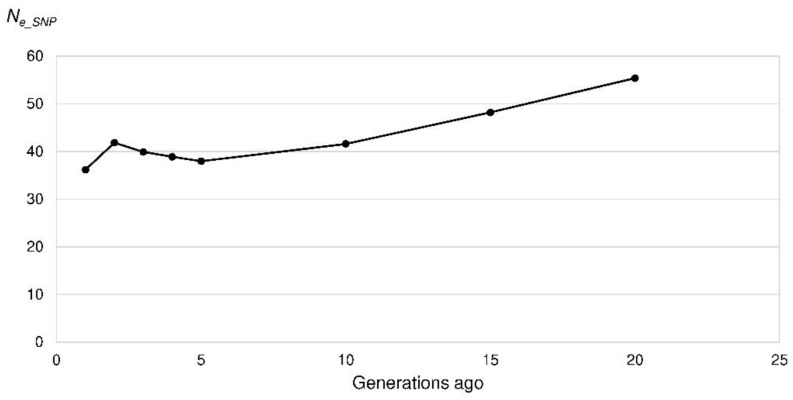
Effective population size (*N_e_SNP_*) of the Polish Lowland Sheepdogs in the last 20 generations based on the linkage disequilibrium among single nucleotide polymorphism (SNP) alleles per chromosome.

**Figure 8:**
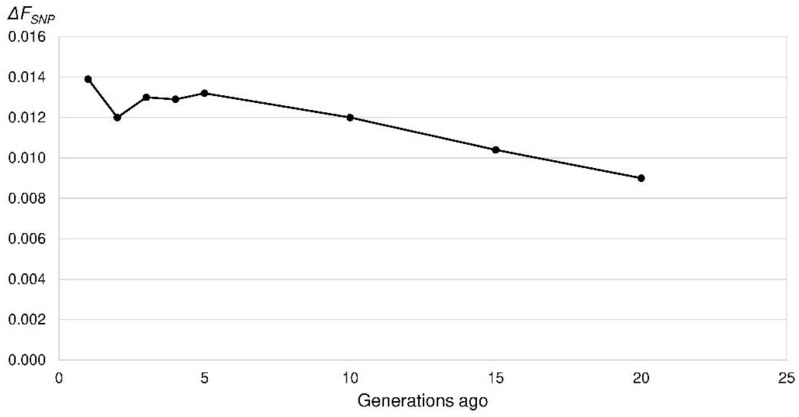
Increase in inbreeding (Δ*F_SNP_*) in the Polish Lowland Sheepdogs in the last 20 generations based on the linkage disequilibrium among single nucleotide polymorphism (SNP) alleles per chromosome.

The mean *r*^2^ as a measure of LD ranged between 0.70 for SNPs 269 bp apart and 0.015 for SNPs 47.5 Mb apart. *N_e_SNP_* was calculated for the last 200 generations, which corresponded to a marker distance of 250 kb to 50 Mb. *N_e_SNP_* decreased from 309 to 101 (50 generations ago) and 38 (five generations ago) (Figure 7). In the very recent generation, the *N_e_SNP_* was 36. From four to two generations ago the *N_e_SNP_* increased to 42. In other words, the increase in inbreeding per generation reached its maximum in the recent generation (Δ*F_SNP_* = 0.014) (Figure 8).

The NetView analysis displayed the PONs as one community (Figure S4). There were no clear clusters within the community. The connections were independent of the inbreeding coefficient.

### 3.3. PANTHER Statistical Overrepresentation Analysis and Functional Classification Test

PANTHER analysis was started with 812 (≥50%), 53 (≥75%), and 12 (≥90%) genes, respectively. Out of these genes, PANTHER could map 743, 48, and 10 genes, respectively. The genes located in 5-SNP ROHs shared by at least 75% of the genotyped PONs (Table S3) were mostly assigned to the biological classes “cellular” and “metabolic processes” (Figure S5). Those shared by at least 50% of the dogs additionally often were assigned to “biological regulation”. The genes in the ROHs shared by at least 90% were assigned to “cellular” and “metabolic processes” and “cellular component organization or biogenesis”. Genes were involved in pathways of angiogenesis, among others (Figure S6). There were no significantly overrepresented or underrepresented gene classes in *ROH5* shared by at least 75%. In those *ROH5* shared by at least 50% of the dogs, genes involved in forelimb morphogenesis were overrepresented among other biological processes (Table S4).

## 4. Discussion

PONs in Germany represent a dog population with a small population size. Anecdotally, all PONs can be traced back to one founder animal in the beginning of pure breeding [7]. Therefore, a low genetic variability and high inbreeding coefficients were expected in this population. Thus, we estimated demographic measures and genetic variability using pedigree and genomic data.

Dogs genotyped were representative for the reference population and thus for the studied PON pedigree population. The mean inbreeding coefficient was slightly lower and *N_e_* slightly higher in the genotyped dogs. This was plausible because we avoided sampling of closely related PONs for genotyping. In addition, the average coancestries of both populations were very similar. Netview analysis showed that the genotyped dogs did not belong to just one line, but reflected many lines.

The *F_Ped_* in the PON population was higher than in other dog breeds [1,2,54]. It was far higher than in the earlier studied Australian PON population [9]. However, that study only contained 12 animals with a mean of 1.7 EqG. Thus, *F_Ped_* was most likely underestimated in that previous study. Important for the high inbreeding coefficient in the PONs studied herein may be the small number of effective founders (Table 2). Especially the proportion of 0.1 effective founders to observed founders was small compared to other breeds. Of 36 Australian populations only the smooth coated Fox terrier and the German shepherd dog had a proportion below the one of the PONs [9].

There were no big differences between the different *F_ROH_* capturing more or less recent inbreeding except for *F_ROH358_*. Thus, α = 0.05 did not seem to be a too strict threshold for the definition of ROHs in the PON population. Despite the high inbreeding coefficient, we did not identify any consensus ROH. It is conceivable that the few effective founders were very distantly related and not inbred that much yet. In addition, breeders avoided line breeding and thus, highly inbred dogs were not mated with closely related dogs. This presumption was supported by NetView analysis that did not display any subpopulations differing in the value of inbreeding coefficients but the amount of inbreeding was equally distributed over the genotyped PONs. The *F_IS_*-values around zero indicated that matings among closely related animals leading to a loss of genetic diversity were not that often in the PON population. The ratios *f_a_*/*f_e_* and *N_g_/f_e_* underlined that there was not that much loss of genetic diversity over the time as in other breeds. Of those studied dog breeds with high known pedigree depth (EqG ≥ 7), only few breeds like the Australian cattle dog or the Lancashire Heeler dog had a higher *f_a_*/*f_e_* ratio than the PONs [9,54,55]. This indicated there were less bottlenecks than in various other breeds. The proportion *N_g_/f_e_* was higher than in PONs only in few breeds (EqG ≥ 7), too [9,55]. Thus, the genetic drift was comparatively small.

Even if an unbalanced use of sires and dams was proved, it did not seem to have much impact on the amount of inbreeding coefficients. Leroy and Baumung [20] reported a proportion of 0.77 between the effective number of reproducers and the total number of reproducers at a random use of reproducers. For the PONs this proportion was 0.35 (sires) and 0.48 (dams), respectively. In the males, those with more than 48 descendants, which corresponded to 13 sires, caused this deviation of 0.77. However, those sires may have been mated to distantly related dams and thus did not influence the inbreeding coefficient significantly.

We expected *F_ROH_* to be higher than *F_Ped_* because of the more precise prediction of the actual proportion of identical by descent (IBD) genome, which related individuals share, even at a large pedigree depth [4,56]. Additionally, the pedigree depth limits the results of pedigree analyses [57]. ROHs cover more generations than those recorded in pedigrees. Because short ROHs capture more ancient inbreeding [57,58], especially the *F_ROH_* based on short ROHs were expected to be higher than *F_Ped_*. However, *F_ROH_* and *F_Ped_* were more similar in the PONs than in various other breeds. The ratio of *F_Ped_* to *F_ROH_* was about 60% in the PONs. In UK Labrador Retrievers it was one-third (100-SNP-*F_ROH_*), in the Lundehund and German Dalmatians it was one-ninth and one-sixth of the 65-SNP-*F_ROH_* [3,28,33]. The reason for these similar values in the PONs was a quite high pedigree completeness. In the Beadchip sample, a pedigree completeness above 90% was found up to the ninth parental generation and mean EqG was 11.2. In the Labrador Retrievers and Dalmatians the mean EqG were 5.6 and 7.5 [3,33]. The completeness and quality of pedigree data is of major importance for the estimation of inbreeding measures using pedigree analysis. An incomplete pedigree can result in underestimated inbreeding measures [17,59,60].

The correlation between *F_ROH_* and *F_Ped_* was in the lower range compared to other studies [3,33,56]. The reason may be the variable depth of branches in the pedigrees. Most PONs had very deep pedigrees. However, several PONs had imbalanced pedigrees. Although the mean completeness of their pedigrees was high with maximal generations of 23, there often were branches in their pedigrees with only very few individuals. Thus, some individuals included in the analysis showed only a few complete generations despite the EqG was in the mean of the PONs. The *F_Ped_* of these individuals was likely to be underestimated. Consequently, for most individuals *F_Ped_* was predicted very well, but there also were several PONs for which *F_Ped_* was underestimated. This may had led to the lower correlation. To prove this hypothesis, the calculation of the correlation between *F_Ped_* and *F_ROH_* was done again without the individuals with less than four, five, six, or seven complete generations. The Pearson correlation coefficient increased to 0.52 and thus was in a similar range compared to previous studies [3,33,56].

As homozygosity increases the incidence of inherited disorders [5], the genes located in most common ROHs were proved to have relevance for health problems in PONs. With *ANGPT1* on CFA 13, a gene located in the ROHs shared by above 90% of the PONs was found, which possibly may be involved in the pathogenesis of PDA, because it is known to be involved in angiogenesis [61]. Furthermore, some previously reported loci for CHD were located in homozygous regions in the PONs [46,47,48,49,50,51,52]. It has to be investigated in further studies, if these loci are also relevant in the progression of CHD in PONs. Genes involved in the forelimb morphogenesis were overrepresented in the ROHs. Thus, those genes were more often observed in homozygous regions in PONs compared to the canine reference genome. This may indicate a predisposition for forelimb diseases like CED. However, there were only reported a few cases of CED as it is like other forelimb diseases are not a very common in the PONs [11]. The gene responsible for furnishing was located in a ROH that was shared by more than 90% of the PONs. This was plausible because all PONs have furnishings by breeding standard.

One factor for high inbreeding coefficients is a small population size. Because of the small choice of possible breeding mates, the probability of matings with related animals is increasing. The $\bar{N_{e}}$ of the PONs ($\bar{N_{e}}$ = 22) was lower than in many other breeds. In two French studies with 50 estimated breeds only five breeds had a $\bar{N_{e}}$ below 50 and of these only the Barbets had a lower $\bar{N_{e}}$ (20) than the PONs. Most breeds had $\bar{N_{e}}$ above 100 with a maximum of 1216 and 2136 in Bulldogs and West Highland White Terriers [1,54]. The *N_e_SNP_* (36) also was low in comparison to various other dog breeds [3,33,62]. Nevertheless the *N_e_SNP_* of the Lundehund (*N_e_SNP_* = 19) was even lower [28]. In various studies critical values of 50 to 100 for the *N_e_* based on pedigree data were given. The threshold value to minimize inbreeding and random genetic impoverishment of *N_e_* = 50 [63] was not exceeded by the PONs. Bijma [64] suggested a Δ*F* of 0.5 to 1.0% per generation. This corresponds to an *N_e_* of 50 to 100. With an expected increase of inbreeding of 22.04% within 50 years the PON population was minimally endangered according to the European Association for Animal Production (EAAP) [65]. Recently the $\bar{N_{e}}$ increased slightly to 29 in 2018. The threshold of 5% increase in inbreeding in 50 years for a not endangered population according to the EAAP would correspond to an *N_e_* of 104 in PONs.

## 5. Conclusions

In conclusion, both the pedigree-based as well as the SNP-based measures of genetic variability showed the same trend. The correlation between *F_Ped_* and *F_ROH_* was in similar range compared to previous studies. The genetic variability in the PON population was small and may be caused by the small number of founder animals at the beginning of pure breeding. Mean inbreeding coefficients of birth year cohorts were on the same level since 1990. However, according to the recommendation of the EAAP the breed had to be classified as minimally endangered. In order to maintain genetic diversity, number of matings for sires should be limited. In case, sires have not more than 48 progeny a popular sire effect can be avoided.

## Figures and Tables

**Table 1:** Overview of the different definitions of runs of homozygosity (ROHs) in this study.

Term for Definition	Thresholds		
	Length (kb)	Minimum SNPs	Maximum Missing SNPs
*ROH5*	60	5	2
*ROH7*	84	7	2
*ROH10*	120	10	2
*ROH20*	240	20	2
*ROH30*	360	30	3
*ROH40*	480	40	4
*ROH50*	600	50	4
*ROH65*	780	65	5
*ROH358*	4300	358	5

Given are the minimum length, the numbers of minimum single nucleotide polymorphisms (SNPs) and the maximum number of missing SNPs in the region for each ROH-definition. Heterozygous SNPs were not allowed in any definition.

**Table 2:** Results of the pedigree-based inbreeding measures calculation for the reference population born from 1990 to 2018 and the genotyped Polish Lowland Sheepdogs. All available pedigree data included 8628 animals.

Inbreeding Measures	Population 1990–2018	Genotyped PONs
Number of animals	4920	153
Mean *F_Ped_*	0.18	0.18
Average coancestry	0.17	0.19
Mean *F_IS_Ped_*	0.011	−0.014
Mean equivalent generations (EqG)	10.09	11.23
Completeness of ancestors in the 5th parental generation	0.93–0.99	0.86–1.00
Mean individual increase in inbreeding (Δ*F_Ped_i_*)	0.023	0.019
Realized effective population size ($\bar{N_{e}}$) (±SE)	22.16 ± 1.46	26.17 ± 0.00
Effective population size by paired increase in coancestry ($\Delta c_{jk}$) (±SE)	–	24.40 ± 0.44
Number of founders (*f*)	103	48
Number of effective founders (*f_e_*)	10	10
Number of ancestors (*a*)	102	29
Number of effective ancestors (*f_a_*)	6	6
Number of ancestors explaining 55% of genetic diversity	2	2
Number of ancestors explaining 75% of genetic diversity	5	5
Founder genome equivalents (*N_g_*)	3.01 ± 0.65	2.81 ± 0.67
Mean generation interval in years (±SE)	4.67 ± 2.05	4.95 ± 1.82

**Table 3:** Overview of the amount and length of runs of homozygosity (ROHs). A ROH was defined in nine different ways. For all definitions the number and length of detected ROHs per individual and detected consensus ROHs are given, respectively. The inbreeding coefficient *F_ROH_* was estimated for all definitions. In total, the genotyped Single Nucleotide Polymorphisms covered 2,201,666,442 bp of the autosomal dog genome.

Definition ROH	Overall Number of ROHs	Length of Shortest ROH (Kb)	Mean Length of ROH (Mb)	Length of Longest ROH (Mb)	Average Cumulative Length of All ROHs (Mb)	Mean *F_ROH_* (and Its Range)
*ROH5*	27,276	61.44	3.76	82.92	669.68	0.30 (0.12–0.46)
*ROH7*	27,274	94.39	3.76	82.92	669.67	0.30 (0.12–0.46)
*ROH10*	27,267	126.14	3.76	82.92	669.67	0.30 (0.12–0.46)
*ROH20*	27,249	241.70	3.76	82.92	669.65	0.30 (0.12–0.46)
*ROH30*	27,042	375.58	3.85	82.92	680.15	0.31 (0.16–0.47)
*ROH40*	27,114	480.66	3.86	82.92	684.87	0.31 (0.17–0.47)
*ROH50*	25,888	600.01	4.02	82.92	679.94	0.31 (0.16–0.47)
*ROH65*	21,391	780.29	4.72	82.92	659.32	0.30 (0.15–0.46)
*ROH358*	6764	4316.64	10.44	82.92	461.34	0.21 (0.00–0.41)

**Table 4:** Overview of the amount and length of runs of homozygosity (ROHs) shared by at least 75% of the genotyped dogs. A ROH was defined in nine different ways. For all definitions the number (*n*) of ROHs, the mean length of detected ROHs and the cumulative length of all ROHs are given.

Definition ROH	*n*	Mean Length (kb)	Cumulative Length of All ROHs (Mb)
*ROH5*	8	731.68	5.85
*ROH7*	8	731.68	5.85
*ROH10*	8	731.68	5.85
*ROH20*	8	731.68	5.85
*ROH30*	8	778.55	6.23
*ROH40*	8	778.50	6.23
*ROH50*	7	813.26	5.69
*ROH65*	4	881.86	3.53
*ROH358*	0	-	-

**Table 5:** Pearson correlation coefficients among inbreeding coefficients. The correlation coefficients with a *p*-value < 0.0001 between the pedigree inbreeding coefficient *F_PED_*, the genomic inbreeding coefficients *F_ROH_* for the different lengths of runs of homozygosity (ROHs), and the fixation index *F_IS_SNP_* are given.

Inbreeding Measures	*F_PED_*	*F_IS_SNP_*	*F_ROH5/7/10/20_*	*F_ROH30_*	*F_ROH40_*	*F_ROH50_*	*F_ROH65_*	*F_ROH358_*
*F_PED_*	1	0.50	0.35	0.39	0.42	0.41	0.39	0.25
*F_IS_SNP_*	0.50	1	0.82	0.89	0.92	0.91	0.89	0.57
*F_ROH5/7/10/20_*	0.35	0.82	1	0.99	0.97	0.98	0.99	0.88
*F_ROH30_*	0.39	0.89	0.99	1	1	1	1	0.82
*F_ROH40_*	0.42	0.92	0.97	1	1	1	1	0.79
*F_ROH50_*	0.41	0.91	0.98	1	1	1	1	0.80
*F_ROH65_*	0.39	0.89	0.99	1	1	1	1	0.83
*F_ROH358_*	0.25	0.57	0.88	0.82	0.79	0.80	0.83	1

**Table 6:** Pearson correlation coefficients between the genomic inbreeding coefficient *F_ROH20_* or *F_ROH50_* and the pedigree inbreeding coefficient *F_Ped_* by number of complete generations. The correlation coefficient with a *p*-value < 0.0001 between *F_ROH20_* or *F_ROH50_* and *F_Ped_* is given. Either all dogs or those with more than three, four, five, or six complete generations in the dataset were regarded in the calculations.

Complete Generations	All	>3	>4	>5	>6
Correlation *F_ROH20_*	0.35	0.37	0.40	0.40	0.46
Correlation *F_ROH50_*	0.41	0.42	0.46	0.46	0.52
*N*	153	151	142	113	89

## Supplementary Materials

The following are available online at https://www.mdpi.com/2076-2615/10/9/1520/s1: Figure S1: Development of the population size of the Polish Lowland Sheepdogs from 1990 to 2018. Given are the number of offsprings and breeding sires and dams per year. Figure S2: Amount of runs of homozygosity (ROH) per chromosome (CFA). The chromosomes are listed from left to right. Figure S3: Incidence-plot of the single nucleotide polymorphisms (SNP) per chromosome. Given is the incidence based on the position of each SNP. Figure S4: Visualization of fine-scale population network for Polish Lowland Sheepdogs. K-NN = 7 (a) and 10 (b). Grey: *F_ROH5_* < 0.16 (3.3%), purple: *F_ROH5_* = 0.16–0.19 (1.3%), turquoise: *F_ROH5_* = 0.19–0.22 (2.0%), dark blue: *F_ROH5_* = 0.22–0.25 (5.3%), dark green: *F_ROH5_* = 0.25–0.28 (17.1%), light green: *F_ROH5_* = 0.28–0.31 (25.0%), orange: *F_ROH5_* = 0.31–0.34 (25.7%), yellow: *F_ROH5_* = 0.34–0.37 (9.9%), brown: *F_ROH5_* = 0.37–0.40 (5.3%), light blue: *F_ROH5_* = 0.40–0.43 (3.9%), pink: *F_ROH5_* ≥ 0.43 (1.3%). Figure S5: PANTHER functional classification analysis. Gene lists of the genes located in *ROH5* shared by at least 75% or 90% of the genotyped Polish Lowland Sheepdogs were analyzed with the functional classification analysis tool of PANTHER. Listed are the biological processes. Figure S6: Results of the functional classification analysis with PANTHER 15.0 for *ROH5* shared by at least 75% or 90% of the genotyped Polish Lowland Sheepdogs. Listed are the pathways. Table S1: Overview of individual results of the analysis of genetic variability in Polish Lowland Sheepdogs. Given are the sex, the year of birth, the number of complete and equivalent (EqG) generations, the inbreeding coefficient (*F_Ped_*) and the increase in inbreeding (Δ*F_Ped_*) based on pedigree data, the total length (in bp) of the runs of homozygosity (ROH) found in the individual and the inbreeding coefficient (*F_ROH20_*) and fixation index (*F_IS_SNP_*) based on genomic data. Table S2: Significances of the differences of the amount of runs of homozygosity per chromosome (CFA). Listed are the *p*-values corrected after Bonferroni and calculated by paired samples Wilcoxon test. Significant (*p* < 0.05) differences are marked in yellow. Table S3: Genes located in ROH5 in Polish Lowland Sheepdogs (PON). Given are the considered dog chromosome (CFA), the start and the end of the region with runs of homozygosity (ROH) on that CFA, the length of the ROH-region in SNPs and all genes located in the ROH-regions. Genes having just an ENSCAFG-number are not listed. For ROH5 shared by at least 50, 75, or 90% of the PONs quantitative trait loci (QTL), detected linkage analyses (LA) or association studies (AS), SNPs and genes already known to be causal or associated with the most breed-relevant diseases canine hip dysplasia (CHD) and progressive retinal atrophy (PRA) or the coat structure of the breed and located in the ROH5 are listed. (a) Includes all ROH5. (b) Includes ROH5 shared by at least 50% of the genotyped PONs. (c) Includes ROH5 shared by at least 75% of the genotyped PONs. (d) Includes ROH5 shared by at least 90% of the genotyped PONs. Table S4: PANTHER statistical overrepresentation test. Gene lists of the genes located in *ROH5* shared by at least 50% or 90% of the genotyped Polish Lowland Sheepdogs were analyzed with the overrepresentation analysis tool of PANTHER. Given are the biological processes the enriched genes are involved in, their fold enrichment and the false discovery rate (*p*-value).
