# Supplementary material for: Genetic Variability in Polish Lowland Sheepdogs Assessed by Pedigree and Genomic Data
Source: Animals (Basel). 2020 Aug 27;10(9):1520. doi: 10.3390/ani10091520 (PMC7552306; doi:10.3390/ani10091520)
Supplement: Supplementary file 1 [file animals-10-01520-s001.zip › TableS4_PANTHER_overrepresentationanalysis.docx]

**Table S4.** PANTHER statistical overrepresentation test. Gene lists of the genes located in ROH5 shared by at least 50% or 90% of the genotyped Polish Lowland Sheepdogs were analyzed with the overrepresentation analysis tool of PANTHER. Given are the biological processes the enriched genes are involved in, their fold enrichment and the false discovery rate (P-value).

| Process | Fold enrichment | P-value |
| --- | --- | --- |
| *ROH5* shared by at least 50% | | |
| Forelimb morphogenesis | 7.35 | 0.025 |
| Calcium-mediated signaling | 3.92 | 0.043 |
| Detection of chemical stimulus involved in sensory perception of smell | < 0.01 | 0.0001 |
| - Sensory perception of smell | 0.10 | 0.003 |
| *ROH5* shared by at least 90% | | |
| Tie signaling pathway | >100 | <0.0001 |
