# Supplementary figures and images for: Genetic Variability in Polish Lowland Sheepdogs Assessed by Pedigree and Genomic Data
Source: Animals (Basel). 2020 Aug 27;10(9):1520. doi: 10.3390/ani10091520 (PMC7552306; doi:10.3390/ani10091520)

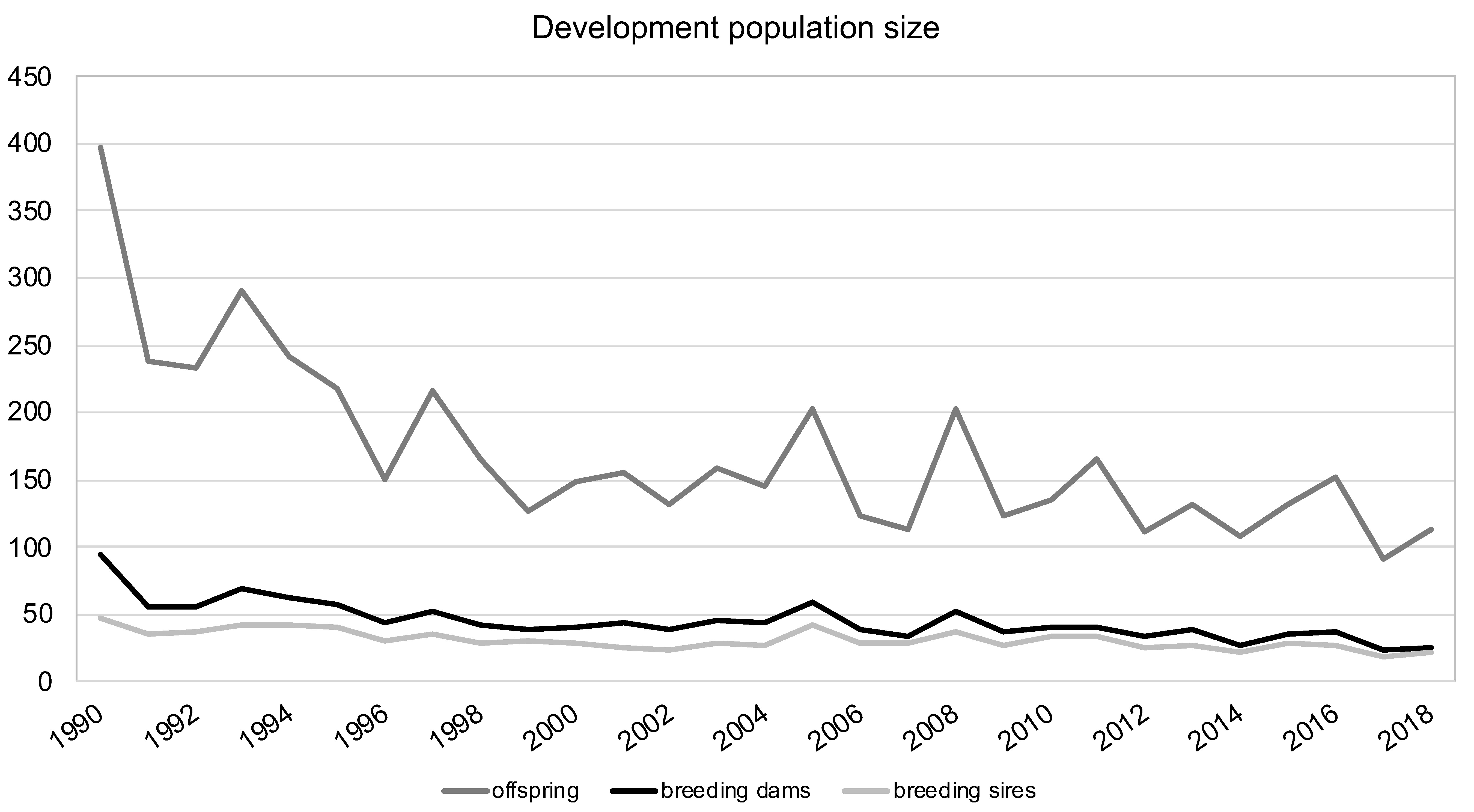

Supplement: Supplementary file 1 [file animals-10-01520-s001.zip › FigureS1_Development_population_size.jpg]

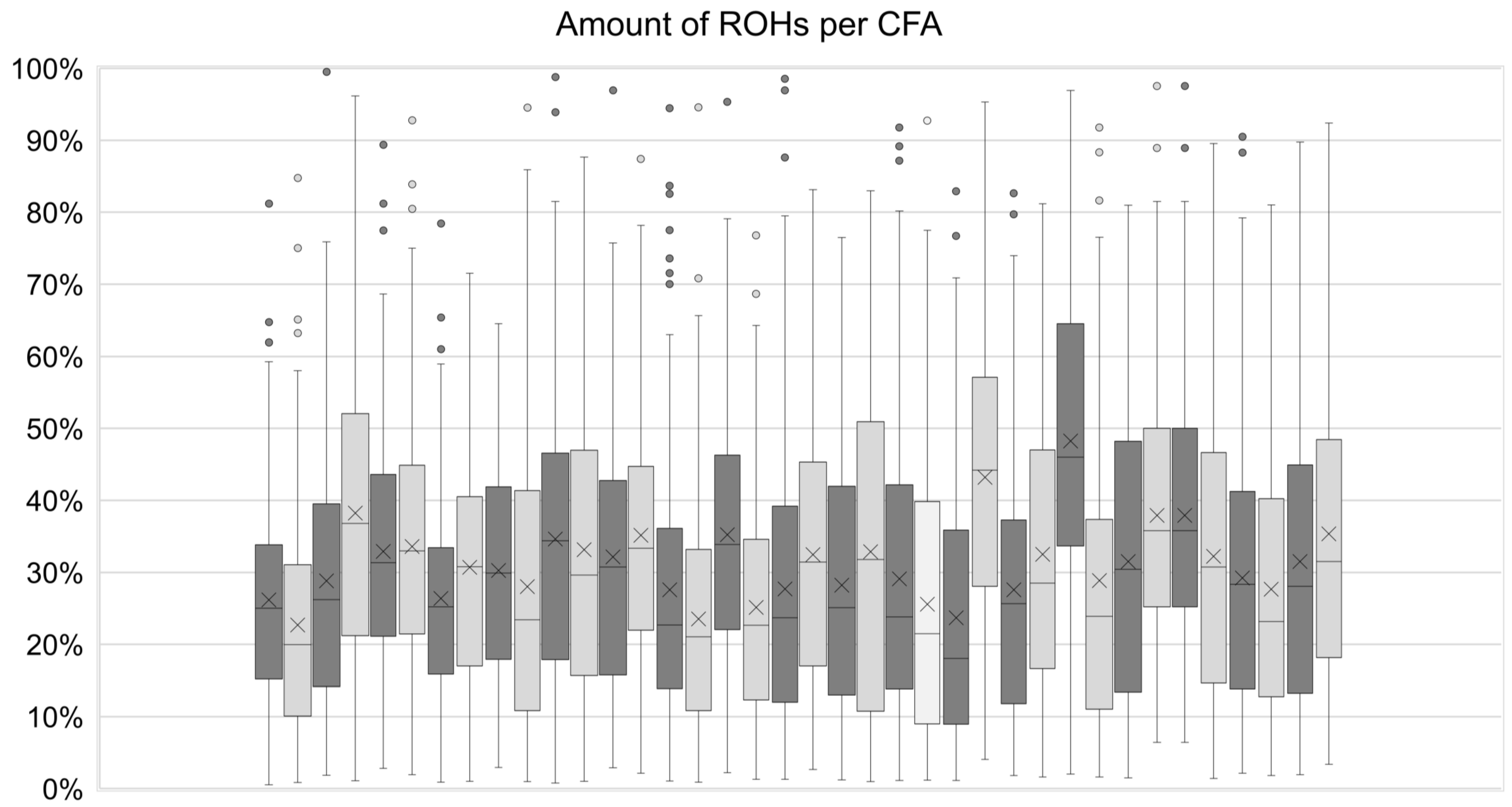

Supplement: Supplementary file 1 [file animals-10-01520-s001.zip › FigureS2_Boxplot_ROH_CFA.jpg]

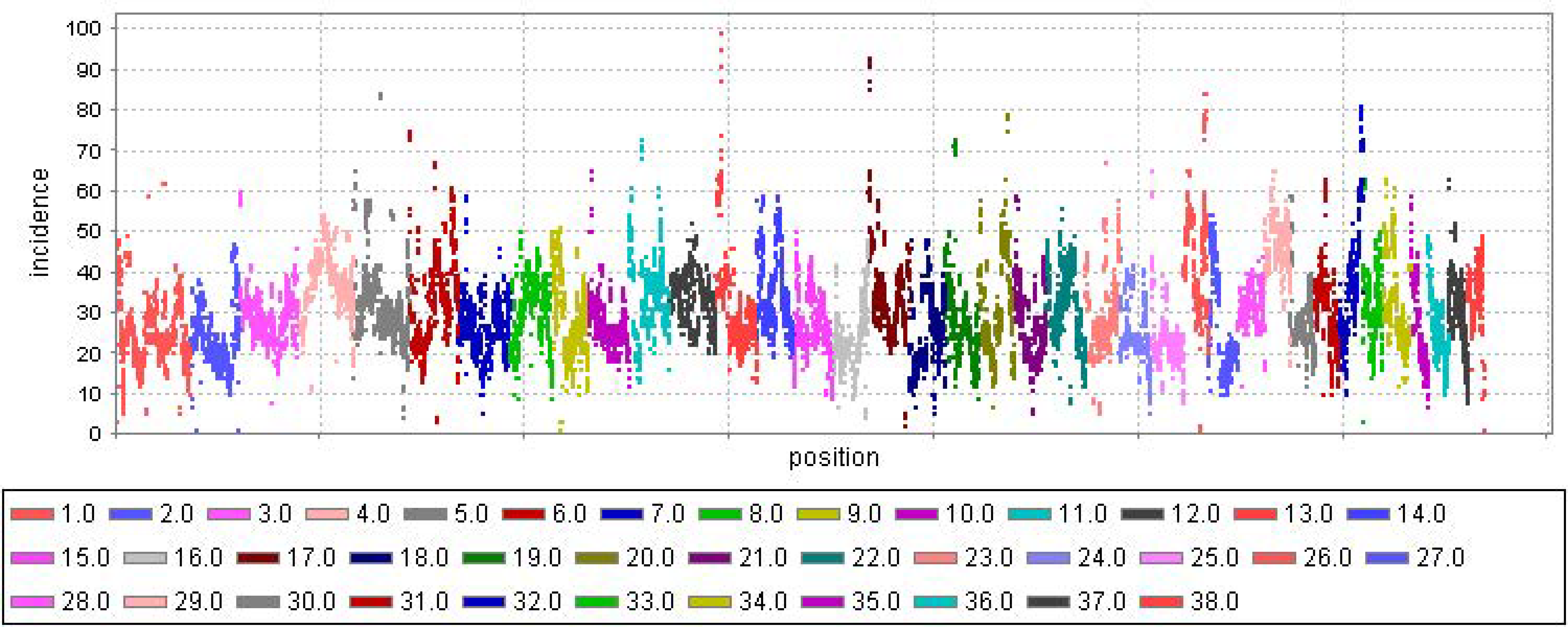

Supplement: Supplementary file 1 [file animals-10-01520-s001.zip › FigureS3_SNP_incidence_CFA_.png]

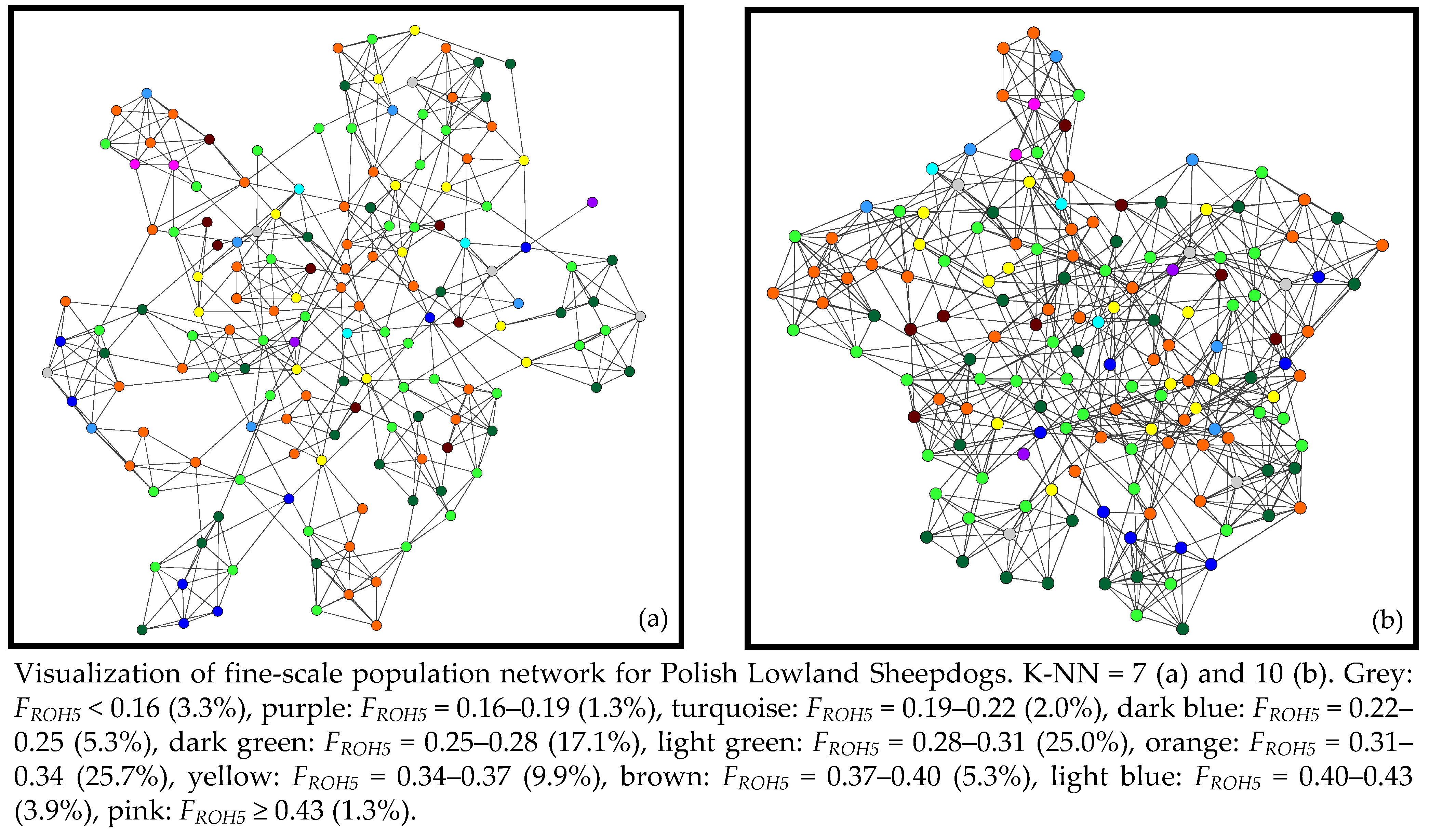

Supplement: Supplementary file 1 [file animals-10-01520-s001.zip › FigureS4_Netview.jpg]

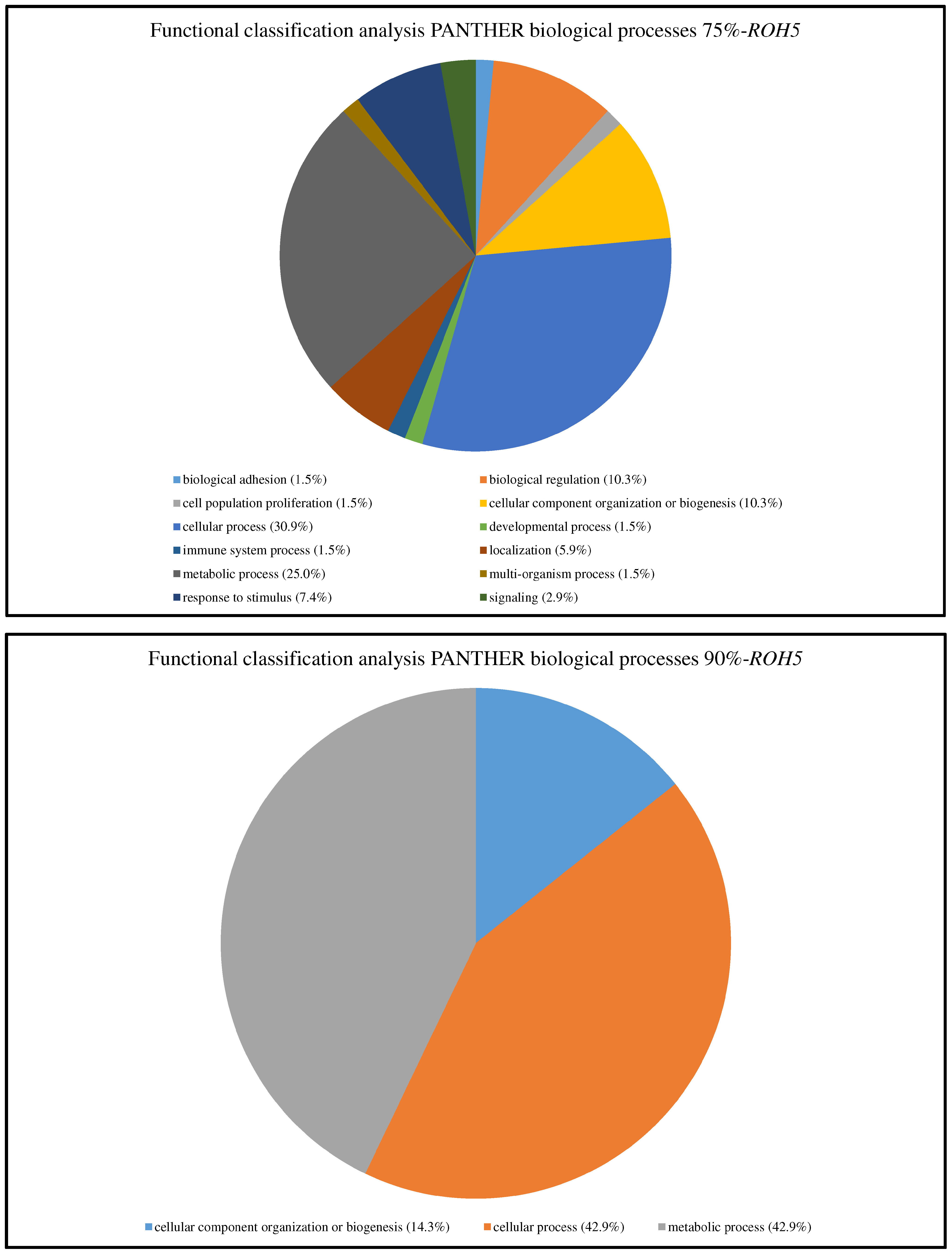

Supplement: Supplementary file 1 [file animals-10-01520-s001.zip › FigureS5_PANTHER_biological_processes.jpg]

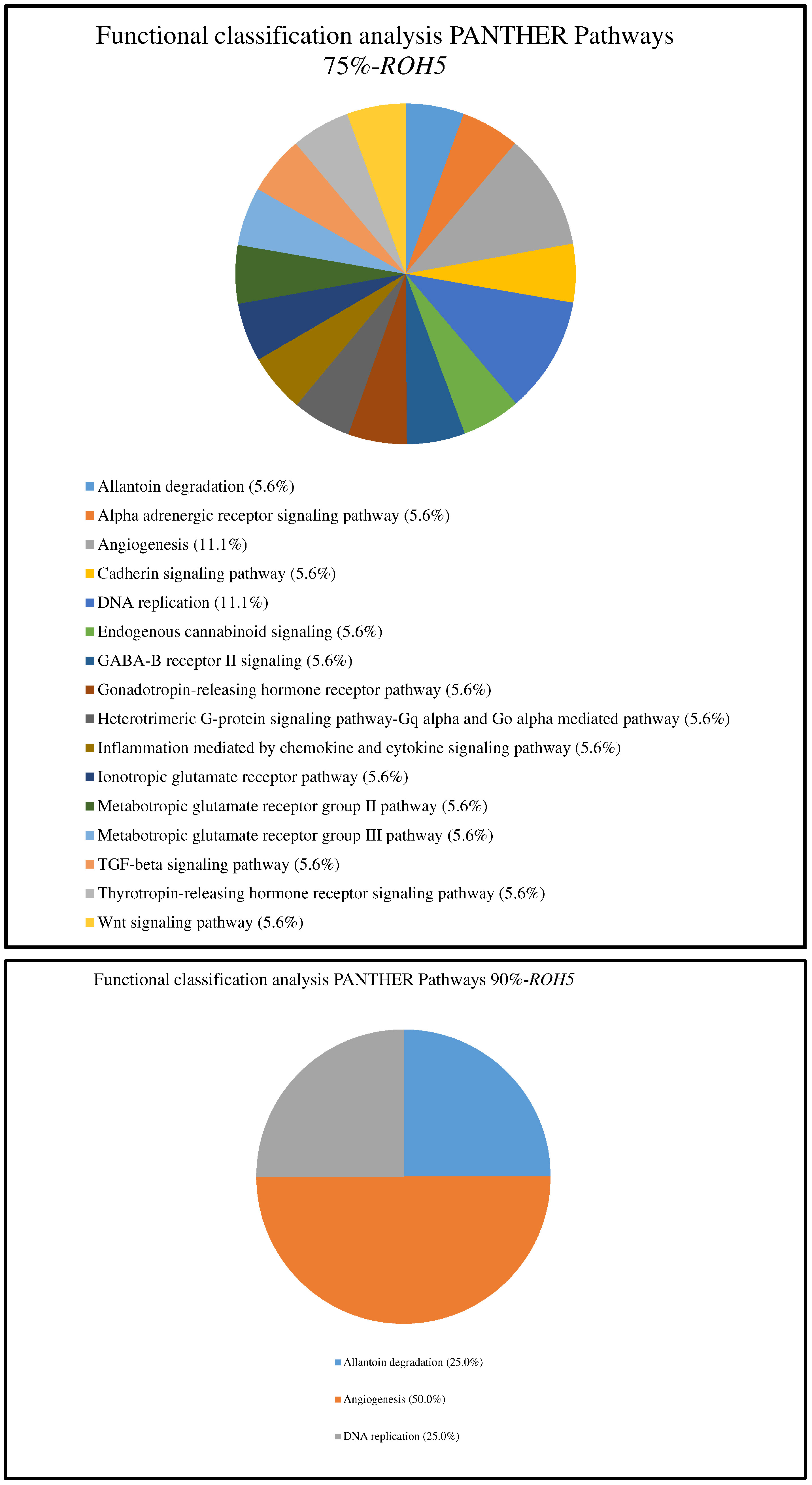

Supplement: Supplementary file 1 [file animals-10-01520-s001.zip › FigureS6_PANTHER_pathways.jpg]
